# Notices to Readers

**Published:** 2014-10-10

## Selected *MMWR* Reports Now Available in French

Selected *MMWR* reports related to the Ebola outbreak and response are now available in French. Beginning this week, the reports can be accessed on the *MMWR* website at http://www.cdc.gov/mmwr. Readers with questions pertaining to reports in French can send them to e-mail, mmwrq@cdc.gov.

## MMWR in Brief Republished in American Journal of Public Health

*MMWR* in Brief is a new feature that provides summaries of serial publications (e.g., Recommendations and Reports, Surveillance Summaries, and Supplements) periodically on the *MMWR* website at http://www.cdc.gov/mmwr/mmwr_briefs.html. The feature was piloted with two postings in November 2013.

Beginning this month, *MMWR* is collaborating with the *American Journal of Public Health* (AJPH), which will republish the *MMWR* in Brief summaries. The first republished summary is for the report, “Outbreaks of Acute Gastroenteritis Transmitted by Person-to-Person Contact — United States, 2009–2010.” That summary was republished online by AJPH on October 8 (available at http://ajph.aphapublications.org/doi/pdf/10.2105/AJPH.2014.302301 ), along with an editorial describing the collaboration (available at http://ajph.aphapublications.org/doi/pdf/10.2105/AJPH.2014.302321 ).

