# Erratum

**Published:** 2014-10-17

## Vol. 63, No. 40

In the Notice to Readers, “*MMWR* in Brief Republished in *American Journal of Public Health*,” on page 908, in the second paragraph, the first Internet link in the third sentence was incorrect. The sentence should read as follows: “That summary was republished online by *AJPH* on October 8 (available at **http://ajph.aphapublications.org/doi/pdf/10.2105/AJPH.2014.10411e13** ), along with an editorial describing the collaboration (available at http://ajph.aphapublications.org/doi/pdf/10.2105/AJPH.2014.302321 ).”

